# Using ctDNA to Inform Adjuvant Therapy for Urologic Malignancies

**DOI:** 10.3390/cancers18071121

**Published:** 2026-03-31

**Authors:** Rajvi Goradia, Taylor Goodstein, Debasish Sundi, Akshay Sood, Shawn Dason, Eric A. Singer

**Affiliations:** 1 Division of Urologic Oncology, The Ohio State University Comprehensive Cancer Center, Columbus, OH 43210, USA; rajvi.goradia@osumc.edu (R.G.); d.sundi@osumc.edu (D.S.); akshay.sood@osumc.edu (A.S.); shawn.dason@osumc.edu (S.D.); 2 Department of Urology, Emory University School of Medicine, Atlanta, GA 30322, USA; tgoodst@emory.edu

**Keywords:** ctDNA, adjuvant therapy, bladder cancer, prostate cancer, kidney cancer

## Abstract

Decisions regarding the use of adjuvant systemic therapy in genitourinary (GU) malignancies—including bladder, kidney, and prostate cancers—are currently driven by clinicopathologic risk factors, which incompletely capture individual risk of residual disease. Consequently, patient selection for adjuvant treatment remains imprecise, leading to both overtreatment of cancers unlikely to recur and undertreatment of those with occult residual disease. Circulating tumor DNA (ctDNA), a minimally invasive liquid biopsy biomarker for minimal residual disease, has emerged as a promising tool to refine adjuvant treatment decision-making. Detection of ctDNA reflects persistent tumor-derived genomic material and often precedes radiographic recurrence, whereas ctDNA negativity is consistently associated with favorable oncologic outcomes. In this review, we summarize the evolving evidence supporting the use of ctDNA to guide adjuvant therapy decisions in bladder, kidney, and prostate cancers. This is not a comprehensive review on all of the potential applications of ctDNA in these malignancies. Rather, we aim to highlight disease-specific, adjuvant-guiding applications, including post-neoadjuvant and post-cystectomy decision-making in bladder cancer and emerging proof-of-concept data in renal cell carcinoma, and explore the potential application of ctDNA in the post-prostatectomy setting. Collectively, these data suggest that ctDNA may enable a paradigm shift toward biologically informed escalation and de-escalation of adjuvant therapy across GU malignancies, while underscoring the need for prospective validation in biomarker-driven clinical trials.

## Simple Summary

This review highlights the emerging role of circulating tumor DNA (ctDNA) as a biomarker to guide adjuvant therapy decisions in genitourinary cancers. Current treatment strategies rely on clinicopathologic features, which do not reliably identify patients with residual disease, leading to both overtreatment and undertreatment. ctDNA offers a noninvasive method to detect minimal residual disease and often identifies recurrence earlier than imaging. Evidence is strongest in bladder cancer, where ctDNA can inform decisions after neoadjuvant therapy and cystectomy. In kidney cancer, its use is limited by low tumor DNA shedding but evolving technologies show promise. In prostate cancer, ctDNA remains investigational but may have future applications post-prostatectomy. Overall, ctDNA has the potential to shift care toward more personalized, biology-driven treatment strategies, though prospective validation is still needed.

## 1. Introduction

Across genitourinary (GU) malignancies, including bladder, kidney and prostate cancers, decisions regarding adjuvant systemic therapy after definitive local treatment are guided primarily by clinicopathologic factors. These factors include tumor stage, grade, nodal involvement, and margin status. Although these factors identify populations at higher risk, they do not reliably determine which individual patients harbor persistent micrometastatic disease.

As a result, current adjuvant strategies are inherently imprecise. Many patients classified as high-risk will never experience recurrence and may be exposed to unnecessary toxicity of additional treatment, while others with occult residual disease may be undertreated by a surveillance-only approach or existing adjuvant monotherapy regimens.

Circulating tumor DNA (ctDNA) has emerged as a promising biomarker to close this gap. ctDNA consists of tumor-derived DNA fragments detectable in the bloodstream, released through tumor cell turnover, and provides a noninvasive measure of minimal residual disease (MRD) after definitive therapy [1,2]. As part of the broader category of liquid biopsy analytes—including circulating tumor cells and other cell-free nucleic acids—ctDNA enables real-time assessment of tumor burden and genomic alterations. Advances in sequencing technologies have improved the sensitivity of ctDNA detection, although performance may vary based on tumor biology, disease burden, and assay platform. Detection of ctDNA reflects ongoing tumor activity and frequently precedes radiographic or clinical recurrence, whereas its absence suggests molecular remission. By directly identifying patients with residual disease, ctDNA offers the potential to refine adjuvant treatment selection, enabling more precise escalation or de-escalation of therapy.

Herein, we provide a concise review of the use of ctDNA to guide adjuvant therapy decisions in prostate, kidney, and urothelial malignancies. This is a narrative review focused on clinically relevant and practice-informing studies. We emphasize prospective clinical trials and selected translational studies that illustrate emerging applications of ctDNA for adjuvant therapy, rather than providing a comprehensive systematic summary of all available data. The available literature is heavily weighted toward recent studies, including early phase trials and emerging prospective datasets, underscoring that ctDNA-guided management remains in a phase of active clinical development rather than established practice.

## 2. Bladder Cancer

Bladder cancer is unique among urothelial cancers for the sheer variety of different applications for which both ctDNA and urinary tumor DNA (utDNA) have the potential to adjudicate adjuvant therapy decisions. In non-muscle–invasive bladder cancer (NMIBC), there is potential use for ctDNA to guide intravesical therapy use after complete transurethral resection of bladder tumor (TURBT). In muscle-invasive bladder cancer (MIBC), ctDNA has been examined as a method of determining pathologic complete response (pCR) after neoadjuvant therapy (NAT) to potentially forgo radical cystectomy (RC). After curative intent treatment, which includes trimodal therapy (TMT, TURBT + chemoradiation) and RC, ctDNA is an attractive tool for guiding adjuvant systemic treatment. We will address each of these applications in the following sections, and a summary of the main trials in this space can be found in Table 1.

### 2.1. ctDNA in NMIBC

NMIBC represents the vast majority of bladder cancer diagnoses (~70%) and is a highly recurrent disease with significant treatment burden [11]. There have been several studies examining the use of ctDNA in NMIBC as a tool for risk stratification and recurrence monitoring, but as of yet, there are no applications for these biomarkers to guide adjuvant therapy decisions—which in this setting consists largely of intravesical treatment [12,13,14,15]. In these studies, ctDNA detection has generally been associated with increased risk of recurrence and may precede clinical or cystoscopic evidence of disease, suggesting potential sensitivity for early detection of residual disease. However, these findings remain exploratory and have not yet translated into changes in intravesical treatment decision-making. Additionally, there is interesting work being conducted utilizing urinary tumor DNA (utDNA), specifically in NMIBC, but this is outside the scope of the current review [16,17].

### 2.2. ctDNA After NAT for MIBC

A high-priority clinical question in bladder cancer is how to define a true CR after systemic NAT that would potentially obviate the need for consolidative treatment such as radical cystectomy. ctDNA after NAT is emerging as a potential tool to guide patient selection for surgery versus observation in MIBC, with ctDNA-negative status strongly associated with pCR and excellent outcomes, while ctDNA-positive status predicts residual disease and poor prognosis.

Several Danish studies have examined this. Dyrskjøt et al. reported that in 68 patients who received neoadjuvant chemotherapy (NAC), ctDNA negativity prior to RC was associated with an 80.7% pCR rate, while none of the ctDNA-positive patients achieved pCR (PPV 100%, NPV 80.8%) [3]. Similarly, another study by Lindskrog et al. found that ctDNA-positivity after NAC significantly predicted disease recurrence (HR 15.2 [5–46.8], *p* < 0.0001) [4]. In this study, ctDNA dynamics during NAC also predicted outcomes, as lack of clearance during treatment was independently associated with RFS when adjusted for pathologic downstaging (HR 4.7, 95% CI 1.2–18.8, *p* = 0.029) [4]. Another Danish study by Christensen et al. analyzed plasma and urine tumor DNA dynamics and demonstrated that both are independently predictive of NAC response, but can also be combined to stratify patients into risk groups (clearance of both vs. clearance of plasma but not urine vs. both remain positive; *p* = 0.003) [5].

The RETAIN-1 trial (NCT02710734) executed a risk-adapted management strategy for patients with MIBC who underwent NAC [18,19]. Pre-NAC TURBT specimens were sequenced for specific mutations in *ATM*, *ERCC2*, *FANCC*, and *RB1*. Patients with one or more mutations and cT0 (determined with repeat TURBT, cytology, and cross-sectional imaging) post-NAC were surveyed rather than undergoing cystectomy (25/70, 36%). The 2-year metastasis-free survival (MFS) in this cohort was 75% (95% CI 54.2–88.4). While this trial did not explicitly use ctDNA (instead harnessing tumor genomic alterations to guide a bladder-sparing approach), it serves as an important proof-of-concept for the risk-adapted management of MIBC. Identifying a subset of patients that can avoid RC or adverse effects of systemic therapy using non-invasive molecular markers remains an unmet need in bladder cancer. To this end, results from the phase II/III NEO-BLAST trial (NCT06537154) are highly anticipated and will provide prospective evidence to support the use of ctDNA as a decision tool for forgoing definitive bladder treatment (either RC or TMT) in patients with MIBC who achieve cCR (determined using ctDNA, utDNA, TURBT, and bladder MRI) after NAC [20,21]. Patients who achieve cCR will be randomized to active surveillance versus definitive bladder therapy. Primary endpoint is 2-year MFS for the phase III component of the trial.

### 2.3. ctDNA After Curative-Intent Treatment for MIBC

#### 2.3.1. ctDNA After Cystectomy

The majority of the evidence for ctDNA in adjudicating adjuvant treatment decisions in bladder cancer is for patients who have received curative-intent treatment in the form of RC. Determining which of these patients are most likely to benefit from adjuvant systemic therapy is of great interest.

Examining ctDNA for this use became highly relevant after a post hoc analysis of the IMvigor010 trial (NCT02450331). This was a multicenter, randomized, phase III trial examining adjuvant atezolizumab versus observation in patients who underwent RC and had ypT2–4a or pT3–4a tumors or were pN+ on final pathology. This study did not meet its primary endpoint of disease-free survival (DFS) difference in the intention-to-treat (ITT) population (median DFS 19.4 months with atezolizumab and 16.6 months with observation [HR 0.89 {95% CI 0.74–1.08}; *p* = 0.24]) [22]. However, in the post hoc analysis, ctDNA was a powerful prognostic as well as predictive marker. ctDNA-positive patients (36% of evaluable samples) had markedly shorter overall survival (OS; HR 6.3 [95% CI 4.3–9.3]) and derived significant benefit from atezolizumab (HR 0.59 [95% CI 0.42–0.83]) [6,7].

The recently published phase III randomized IMvigor011 trial was designed with these retrospective findings in mind [8]. Serial ctDNA testing was performed for up to one year in 761 patients who underwent RC for MIBC and had no radiographic evidence of disease. Patients who became ctDNA-positive during surveillance (*n* = 250) were randomly assigned to receive atezolizumab (*n* = 167) or placebo (*n* = 83). The primary end point was DFS and the secondary endpoint was OS. Median DFS was 9.9 vs. 4.8 months (HR 0.64, 95% CI 0.47–0.87, *p* = 0.005) and median OS was 32.8 vs. 21.1 months (HR 0.59, 95% CI 0.39–0.9, *p* = 0.01) with atezolizumab vs. placebo, respectively. Among 357 persistently ctDNA-negative patients, 1-year DFS was 95% and 2-year DFS was 88% [8]. Thus, the IMvigor011 trial strongly supports the use of post-RC ctDNA to guide secondary systemic immune-checkpoint blockade among patients with MIBC who have undergone RC.

It is notable that two different adjuvant immunotherapy trials, Checkmate-274 (adjuvant nivolumab) and AMBASSADOR (adjuvant pembrolizumab), demonstrated DFS benefit without stratifying patients based on ctDNA status [23,24]. These agents are currently FDA approved for specific high-risk patients (ypT2 or higher, pT3 or higher, pN+, or ypN+) following RC. In the recently published 5-year updated efficacy results of CheckMate 274, however, a post hoc analysis stratified by ctDNA positivity was performed in the subset of patients for whom these data were available (54/133, 40.6%). In patients with detectable ctDNA, nivolumab was associated with a median DFS HR of 0.35 (95% CI 0.18–0.66) compared to placebo. In patients with an undetectable ctDNA, there was no association of nivolumab (versus placebo) with DFS (median DFS HR 0.99; 95% CI 0.51–1.93) [9]. These exploratory findings will require prospective validation.

The TOMBOLA trial (NCT04138628), a Danish phase II trial evaluating serial ctDNA testing to guide postoperative immunotherapy in patients with MIBC treated with NAC and RC, has added further context to our understanding of ctDNA in patients with MIBC [10]. Patients with positive postoperative ctDNA were initiated on atezolizumab, and patients who were ctDNA negative received salvage immunotherapy only with radiographic evidence of metastases. The study classified patients as clinically high or low risk (high-risk ypT2 or higher and/or N+ disease at RC) and monitored ctDNA postoperatively. Of the patients who were or became ctDNA positive, 50% were low risk, and would not have ordinarily been eligible for adjuvant immunotherapy under current guideline criteria [25]. Notably, no high-risk, ctDNA-negative patients (30% of ctDNA-negative patients) developed metastatic recurrence during the follow-up period. Upon initiation of atezolizumab, ctDNA clearance was associated with improved RFS in both high- and low-risk patients (HR 2.7 [95% CI 1.1–6.6], *p* = 0.025 and HR 11.7 [95% CI 1.3–104.8], *p* = 0.005, respectively).

The MODERN trial (NCT05987241) is an ongoing phase II/III study evaluating whether ctDNA can guide use of adjuvant nivolumab after surgical resection for urothelial cancer [26]. Patients with detectable ctDNA post-operatively are randomized to receive adjuvant nivolumab alone versus nivolumab plus relatlimab (inhibitor of the immune checkpoint LAG-3), while those without detectable ctDNA are randomized to adjuvant nivolumab versus surveillance. This trial is uniquely designed to determine whether patients with detectable ctDNA specifically benefit from an escalation of therapy, and whether patients without detectable ctDNA might still benefit from adjuvant treatment at all.

#### 2.3.2. ctDNA After Trimodality Therapy

There is not an established use for adjuvant therapy following TMT in MIBC. An ongoing trial examining this is the phase II, single-arm NEXT trial (NCT0317102), which is assessing adjuvant nivolumab following chemoradiation in localized MIBC [27]. ctDNA monitoring is not a part of this study’s protocol. In fact, using ctDNA as a decision aid to guide treatment in patients undergoing TMT has not yet been investigated. However, there have been retrospective studies demonstrating that ctDNA dynamics correlate with clinical outcomes following TMT [28,29].

In conclusion, there are a variety of disease states within bladder cancer that could potentially be revolutionized by ctDNA or utDNA. The areas with the most robust evidence include the use of ctDNA after RC and the use of ctDNA after NAC. Additional research is warranted to examine the application of ctDNA or utDNA to adjudicate adjuvant therapy in NMIBC or patients treated by TMT.

## 3. Kidney Cancer

Results from adjuvant clinical trials in renal cell carcinoma (RCC) have been mixed. The only positive trial leading to FDA approval has been KEYNOTE 564, which demonstrated improved OS and RFS with adjuvant pembrolizumab in patients with clear-cell RCC [30,31]. ctDNA measurement was not included in this study. The RAMPART trial demonstrated improved DFS with adjuvant durvalumab plus tremelimumab at 2 years in patients with resected RCC at high- or intermediate-risk of relapse (84% vs. 78% in the active monitoring group; *p* = 0.009) [32]. In a secondary analysis stratified by risk group, high-risk patients (*n* = 311) benefited the most from adjuvant treatment (HR 0.52 [95% CI: 0.34–0.80], *p* = 0.002). A translational study arm for this trial, TransRAMPART, is currently underway, with a focus on prospective, longitudinal collection of blood, urine, and tissue samples to identify new biomarkers and therapeutic targets for patients with localized RCC [33]. Negative trials in this space have included IMmotion010 (adjuvant atezolizumab), Checkmate-914 (adjuvant nivolumab ± ipilimumab), and PROSPER RCC (perioperative nivolumab) [34,35,36]. Results from LITESPARK-022, investigating the combination of the HIF-2a inhibitor belzutifan with pembrolizumab vs. pembrolizumab alone in the adjuvant setting for clear cell RCC patients, are highly anticipated [37].

One proposed explanation for the mixed results of adjuvant trials in RCC is the lack of reliable biomarkers to guide treatment selection. While ctDNA has demonstrated clinical utility in detecting MRD in bladder cancer, its role in RCC remains less well defined. A key limitation is the relatively low level of ctDNA detected in patients with RCC, with one series reporting detectable ctDNA in only approximately 60% of patients with metastatic disease [38].

Several biological factors may contribute to this phenomenon, including lower rates of tumor cell turnover and a comparatively lower mutational burden. In addition, the tumor microenvironment in RCC—characterized by hypoxia and the presence of a pseudocapsule—may further limit the release of tumor DNA into the circulation. Interestingly, even in cases where tumors extend directly into the venous system, ctDNA detection remains inconsistent. In the NAXIVA study, for example, ctDNA was detectable in only 25% of patients with venous tumor thrombus [39]. This finding suggests that low ctDNA yield in RCC is not solely explained by anatomic barriers and highlights an important biological limitation of this biomarker in this disease.

Emerging approaches aim to improve ctDNA detection in RCC. Methods that assess DNA methylation patterns, rather than relying solely on mutation detection, have shown improved sensitivity in small studies [40,41]. Similarly, tumor-informed assays—designed using genomic information from an individual patient’s tumor—may enhance detection rates compared to untargeted approaches [42]. However, these findings are largely based on early phase or small cohort studies. For example, in the DIAMOND study, tumor-informed approaches demonstrated higher detection rates than standard methods, though sensitivity remained modest overall [42]. Likewise, a pilot study of 12 patients suggested that ctDNA dynamics may correlate with treatment response, but the small sample size limits the strength of these conclusions [43].

Additional techniques, including analysis of DNA fragmentation patterns and broader genomic profiling approaches, have also been explored across multiple cancer types, including RCC [44,45]. While these methods show promise in improving detection performance, their clinical applicability in RCC specifically remains uncertain. Finally, genomic profiling studies have identified associations between certain mutations and clinical outcomes; however, these findings are often based on small subgroups and wide confidence intervals, underscoring the need for cautious interpretation [46].

Taken together, although advances in ctDNA detection methods may improve sensitivity in RCC, current evidence remains preliminary. Larger, prospective studies are needed to determine whether these approaches can meaningfully inform clinical decision-making in this disease.

Most studies examining the use of ctDNA in RCC have been performed in the metastatic setting, with mostly small, retrospective cohorts examining a potential prognostic use for ctDNA in localized disease [42,47,48,49]. Regarding adjuvant therapy decisions, ctDNA has potential applications in patients who have undergone extirpative surgery (either partial or radical nephrectomy, including M1 with no evidence of disease [NED]), tumor ablation, or stereotactic ablative radiotherapy (SABR). We will address each of these in the sections below.

### 3.1. ctDNA After Extirpative Surgery

To date, there are no clinical trials that specifically use ctDNA to guide adjuvant therapy decisions in kidney cancer. Most of the current literature focuses on advanced RCC or retrospective evaluations of ctDNA in determining prognosis. For example, a retrospective analysis by Correa et al. analyzed 45 RCC patients who underwent nephrectomy and had banked ctDNA specimens using the Signatera (Natera, Inc., Austin, TX, USA) tumor-informed 16-plex PCR assay [47]. They found that ctDNA positivity at any time point had a sensitivity of relapse prediction of 84% and a PPV of 90%. A post-operative positive ctDNA demonstrated a PPV of 100%. Another study, by Büttner et al., looked at *SHOX2* hypermethylation (mSHOX2) in ctDNA of 45 patients after nephrectomy. A total of 17 (37.8%) were positive and the presence of mSHOX2 was an independent risk factor for reduced OS (HR 2.94 [95%CI: 1.09–7.90], *p* = 0.03) and MFS (HR 4.51 [95%CI:1.05–19], *p* = 0.04) [50].

An ongoing prospective study, MRD-GATE RCC, has reported preliminary data on the use of ultrasensitive ctDNA assays in 27 patients with localized RCC who underwent extirpative surgery [51]. Four patients (15.4%) were ctDNA positive soon after surgery, and cancer recurred in three of these patients (at 545, 188, and 159 days from ctDNA positivity). Of the 22 remaining patients, 21 were ctDNA negative and showed no signs of recurrence at a median follow up of 7.9 months.

These studies show an increasing role for ctDNA in determining prognosis in RCC, but for guiding the selection of adjuvant therapy, a recently published case series (*n* = 6 with different RCC variants) offered a tantalizing glimpse of how this might be applied [48]. In one patient (M1 NED), ctDNA was used to monitor disease status while on a treatment break from adjuvant pembrolizumab (due to a severe immune-related adverse event). ctDNA became detectable and treatment was reinitiated at a lower dose, resulting in a subsequent clearance of ctDNA. To date, this patient has had no imaging-detected disease recurrence. A second patient in the same series was started on adjuvant doxorubicin for pT4N0 malignant angiomyolipoma. ctDNA levels initially decreased but then plateaued. A specific TSC2 mutation was detected in the ctDNA, and this prompted a change in therapy to everolimus, leading to undetectable ctDNA in one month. This patient remained recurrence free for a year and stopped therapy. During surveillance, ctDNA positivity in the setting of indeterminate pulmonary micronodules led to reinitiation of therapy. Although limited by sample size, this study establishes an important proof of concept and generates hypotheses for how ctDNA may ultimately inform adjuvant therapy selection in localized RCC.

### 3.2. ctDNA After Tumor Ablation or SABR

There is currently no established paradigm for the use of adjuvant therapy or ctDNA monitoring after tumor ablation or SABR. Existing studies examining the use of ctDNA are available for SABR only. In a study of 17 patients with oligometastatic RCC undergoing SABR after nephrectomy, the sensitivity, specificity, PPV and NPV of ctDNA to detect progression were 65%, 100%, 100% and 81%, respectively [52]. One out of the seven patients who progressed started systemic therapy with interleukin-2, which was followed by undetectable ctDNA at completion of therapy. This study suggests a role for ctDNA in detecting disease recurrence in patients with radiated kidney tumors, but what that role is and how it might inform a potential adjuvant therapy is currently unknown.

## 4. Prostate Cancer

Currently, use of ctDNA in prostate cancer is limited due to the absence of detectable ctDNA and mutations, especially for patients with localized disease [53]. A prospective, multi-center study from five high-volume centers found that baseline ctDNA positivity had a significantly shorter time to biochemical recurrence (HR 3.64, 95% CI 2.23–5.93, *p* < 0.001) compared to ctDNA negativity in 628 men with intermediate- and high-risk prostate cancer [54]. Similar findings from other international cohorts have been reported for biochemical recurrence (BCR) [55,56].

From a clinical standpoint, the post-prostatectomy setting may represent the most biologically and clinically rational context in which ctDNA could inform adjuvant treatment decisions in prostate cancer.

### ctDNA to Guide Adjuvant Radiation Following Prostatectomy

Based on the results of three multicenter, practice-changing randomized controlled trials (RADICALS-RT, GETUG AFU 17, and RAVES 2) and the ARTISTIC meta-analyses, the use of adjuvant radiotherapy for high-risk patients post prostatectomy has been largely replaced by early salvage radiotherapy [57,58,59,60]. Early salvage is dependent on prostate specific antigen (PSA) detection (at a threshold of 0.1 or 0.2 ng/dL) [57,58,59]. A meta-analysis of three clinical trials (GETUG-AFU-16; NRG/RTOG-9601, EORTC-22911) found that a PSA value of >0.5 at the start of salvage therapy was an independent prognostic factor for worse PFS, MFS and OS [61]. Whether or not ctDNA could potentially improve the sensitivity/specificity of PSA in this disease setting, or re-establish a role for adjuvant radiotherapy, is currently not established [62].

Though not a direct correlate, ORIOLE is a phase II randomized controlled trial assessing the role of SABR in oligometastatic prostate cancer [63]. At baseline, quantitative and qualitative measurements of ctDNA were performed. There were no differences in concentrations of baseline ctDNA allele fractions between patients that progressed vs. those that did not in either the SABR (*n* = 32) or observation arm (*n* = 18). However, in a sub-analysis of 22 patients stratified by detectable ctDNA and truncating mutations in high-risk genes, progression-free survival (PFS) was significantly longer in the SABR arm vs. the observation arm in patients without high-risk mutations (*n* = 15) but not in patients harboring said mutations (*n* = 7). A reasonable hypothesis to draw from this finding is that the use of ctDNA in prostate cancer may be limited to tumor-informed ctDNA. In other words, the mutational landscape of each individual patient may matter when determining whether radiotherapy can be of benefit. A small study from 2015 examining 55 patients undergoing adjuvant vs. salvage radiation found that detection of circulating tumor cells (CTC) at any timepoint (*n* = 16) was associated with significantly shorter time to BCR compared to CTC– patients at all timepoints (*n* = 38, *p* = 0.04) [64]. Further, baseline CTC+ patients with extracapsular extension and/or seminal vesicle invasion had significantly shorter time to BCR compared to CTC– patients with the same.

In summary, though the use of ctDNA in prostate cancer is not currently used to guide recurrence risk and is certainly not established as a tool to guide adjuvant therapy, there is some limited evidence that opens the door to this possibility.

## 5. Future Directions

As results from prospective, large-scale studies and clinical trials continue to provide evidence, ctDNA is poised to play a key role in the detection of MRD for urological cancers [51,65,66]. With the increased sensitivity of novel techniques, tumor-informed approaches and application of epigenomic assays, even tumors with historically low ctDNA yield may show promise. The use of newer, more sensitive ctDNA detection methods in prospective studies and clinical trials may lead to wider adoption of ctDNA in RCC [33,65,67]. Novel trial designs for advanced and localized cancers have begun to incorporate ctDNA as a biomarker to guide treatment intensification and de-escalation [68,69]. Another advantage in urological malignancies has been the availability of urine as a medium to identify novel circulating markers. utDNA is currently being investigated in BCG-unresponsive NMIBC patients and has high potential to aid in risk stratification (NCT07187063) [16]. Ongoing clinical trials utilizing ctDNA in GU malignancies can be found in Table 2.

## 6. Conclusions

ctDNA has the potential to shift adjuvant therapy decision-making in genitourinary malignancies from clinicopathologic risk stratification toward biologically defined residual disease. Evidence is most mature in bladder cancer, where ctDNA-guided strategies are already reshaping long-held standards. In kidney and prostate cancers, emerging tumor-informed approaches provide an early proof of concept, despite ongoing technical and biologic challenges. As prospective ctDNA-driven trials mature, ctDNA may ultimately enable a more precise, individualized approach to adjuvant therapy across GU cancers.

## Figures and Tables

**Table 1:** ctDNA in bladder cancer: summary of key studies.

Author	Design/Phase	Population (N)	ctDNA Assessment	Outcomes	Key ctDNA Findings
Dyrskjøt et al. [3]	Prospective cohort	MIBC (68)	Pre and post-NAC (pre-RC)	pCR	100% PPV, 81% NPV for detecting pCR post-NAC ctDNA+ pts who did not achieve pCR had worse RFS and OS than ctDNA−
Lindskrog et al. [4]	Prospective observational	MIBC post-NAC (68); NAC-naïve (102)	Long-term serial during/after NAC; before and after RC in NAC-naïve	Prognostic value of ctDNA post-RC	ctDNA strongly prognostic for recurrence (HR 15.2) and OS in NAC-treated and naïve patients
Christensen et al. [5]	Prospective observational	MIBC post-NAC (92)	Serial plasma + cell-free urine DNA	Recurrence risk	Combined plasma/urine dynamics improved risk stratification; dual clearance showed best recurrence outcomes
IMvigor010 [6,7]	Phase III, post hoc analysis	Post-RC high-risk MIBC (581)	Post-RC	OS in BEP	ctDNA+ pts had worse OS (HR: 6.3) compared to ctDNA-pts, & improved response to atezolizumab (HR: 0.59) compared to observation
IMvigor011 [8]	Phase III	ctDNA+ Post-RC MIBC (250)	Serial up to 1 year	DFS, OS	Pts in the atezo group had significantly better DFS (HR: 0.64) and OS (HR: 0.59) compared to placebo
CheckMate 274 [9]	Phase III, post hoc analysis	Post-RC high-risk MIBC (133)	Post-RC	DFS, OS	Benefit of nivolumab confined to ctDNA+; no clear benefit in ctDNA
TOMBOLA [10]	Phase II	MIBC post-NAC + RC (178)	Serial post-RC	RFS	ctDNA status is prognostic for recurrence especially in high-risk; clearance associated with improved RFS

Abbreviations: ctDNA = circulating tumor DNA; MIBC = muscle-invasive bladder cancer; NAC = neoadjuvant chemotherapy; RC = radical cystectomy; pCR = pathologic complete response; DFS = disease-free survival; RFS = recurrence-free survival; OS = overall survival; BEP = biomarker-evaluable population. Notes: ctDNA− = undetectable ctDNA; ctDNA+ = detectable ctDNA. Post hoc analyses were not prespecified and should be interpreted as exploratory.

**Table 2:** Summary of current GU prospective trials and studies utilizing ctDNA as an endpoint.

	ctDNA Role
Bladder	
MODERN: An Integrated Phase II/III and Phase III Trial of MRD-Based Optimization of ADjuvant ThErapy in URothelial CaNcer (NCT05987241) [26]	ctDNA kinetics to guide adjuvant nivolumab + relatlimab
RAD-SG: Adaptive RADiation Therapy with Concurrent Sacituzumab Govitecan (SG) for Bladder Preservation in Patients with MIBC (NCT05833867) [70]	To identify novel predictive biomarkers for response to radiation and immunotherapy
Kidney	
MRD-GATE RCC: Molecular Residual Disease (MRD) Guided Adjuvant ThErapy in Renal Cell Carcinoma (RCC) (NCT06005818) [67]	To guide use of adjuvant pembrolizumab based on MRD estimation with ctDNA
KIDNEY-PAGER: Analysis of Circulating Tumor DNA as a Biomarker in Renal Cancer—an Observational Trial (NCT06145139) [65]	Response monitoring, risk stratification
Prostate	
PROTRACT: PROstate Cancer TReatment Optimization Via Analysis of Circulating Tumour DNA (NCT04015622) [71]	ctDNA fraction-guided treatment with Enzalutamide or Docetaxel after Abiraterone
Rare GU tumors	
SMART: A Phase II Study of Sacituzumab Govitecan With or Without Atezolizumab Immunotherapy in Rare Genitourinary Tumors (NCT06161532) [72]	Response monitoring

## Data Availability

No new data were created or analyzed in this study.

## Glossary

ctDNA	circulating tumor DNA
cfDNA	cell-free DNA
MRD	minimal residual disease
GU	genitourinary
NMIBC	non-muscle-invasive bladder cancer
MIBC	muscle-invasive bladder cancer
TURBT	transurethral resection of bladder tumor
NAC	neoadjuvant chemotherapy
RC	radical cystectomy
TMT	trimodality therapy
pCR	pathologic complete response
cCR	clinical complete response
DFS	disease-free survival
RFS	recurrence-free survival
MFS	metastasis-free survival
OS	overall survival
HR	hazard ratio
CI	confidence interval
PPV	positive predictive value
NPV	negative predictive value
BEP	biomarker-evaluable population
DDR	DNA damage repair
RCC	renal cell carcinoma
SABR	stereotactic ablative radiotherapy
M1 NED	metastatic disease with no evidence of disease
BCR	biochemical recurrence
PSA	prostate-specific antigen
PFS	progression-free survival
CTC	circulating tumor cell
utDNA	urinary tumor DNA
